# Cell Free Bacteriophage Synthesis from Engineered Strains Improves Yield

**DOI:** 10.1021/acssynbio.3c00239

**Published:** 2023-08-07

**Authors:** Rani Brooks, Lisa Morici, Nicholas Sandoval

**Affiliations:** † Interdisciplinary Bioinnovation PhD Program, Tulane University, New Orleans, Louisiana 70118-5665, United States; ‡ Department of Microbiology and Immunology, Tulane University School of Medicine, New Orleans, Louisiana 70112, United States; § Department of Chemical and Biomolecular Engineering, Tulane University, New Orleans, Louisiana 70118, United States

**Keywords:** cell-free expression systems, cell-free bacteriophage synthesis (CFBS), T7, CRISRPi, gene expression, TX-TL

## Abstract

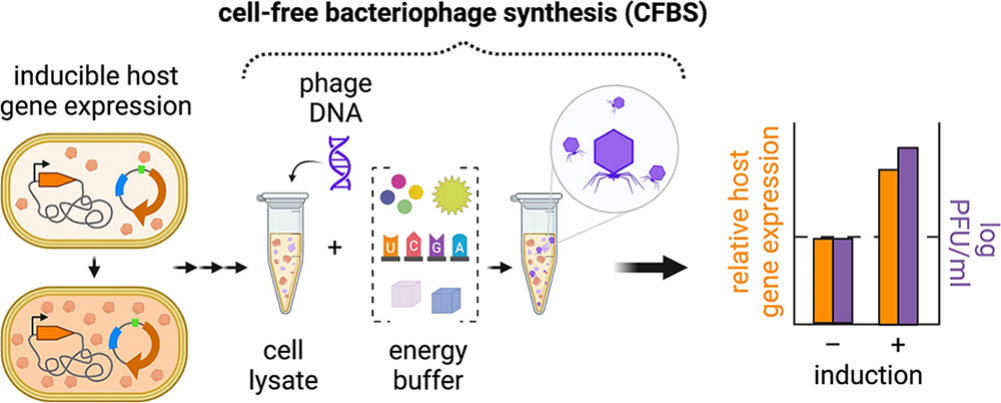

Phage therapy to treat life-threatening drug-resistant infections has been hampered by technical challenges in phage production. Cell-free bacteriophage synthesis (CFBS) can overcome the limitations of standard phage production methods by manufacturing phage virions in vitro. CFBS mimics intracellular phage assembly using transcription/translation machinery (TXTL) harvested from bacterial lysates and combined with reagents to synthesize proteins encoded by a phage genomic DNA template. These systems may enable rapid phage production and engineering to accelerate phages from bench-to-bedside. TXTL harvested from wild type or commonly used bacterial strains was not optimized for bacteriophage production. Here, we demonstrate that TXTL from genetically modified *E. coli* BL21 can be used to enhance phage T7 yields in vitro by CFBS. Expression of 18 *E. coli* BL21 genes was manipulated by inducible CRISPR interference (CRISPRi) mediated by nuclease deficient Cas12a from *F. novicida* (d*Fn*Cas12a) to identify genes implicated in T7 propagation as positive or negative effectors. Genes shown to have a significant effect were overexpressed (positive effectors) or repressed (negative effectors) to modify the genetic background of TXTL harvested for CFBS. Phage T7 CFBS yields were improved by up to 10-fold in vitro through overexpression of translation initiation factor IF-3 (*infC*) and small RNAs OxyS and CyaR and by repression of RecC subunit exonuclease RecBCD. Continued improvement of CFBS will mitigate phage manufacturing bottlenecks and lower hurdles to widespread adoption of phage therapy.

## Introduction

Multidrug-resistant (MDR) bacterial infections pose one of the greatest emerging public health threats. MDR bacteria possess both intrinsic and acquired mechanisms of resistance to most commonly used antibiotics (e.g., small molecules), leaving few treatment options for the most at-risk patients. The Centers for Disease Control reports 2.8 million MDR infections per year with 35,000 deaths.^1^ The World Health Organization has predicted that by the year 2050, deaths due to MDR infections will surpass all other causes of deaths. It is therefore imperative to develop new therapeutic strategies to combat MDR bacteria.

Phage therapy, a resurgent antibacterial treatment, has the potential to cure bacterial infections resistant to small molecule antibiotics. Bacteriophage, or “phage,” are ubiquitous viruses that can be enriched from environmental samples (e.g., water, sewage, soil) or engineered in laboratories.^2,3^ Phage therapy is a form of personalized medicine that takes advantage of the high precision of phages to prey upon specific hosts to clear infection-causing bacteria. Unlike small molecule antibiotics, phage do not harm protective flora, which leads to increased susceptibility to future infections.^4^

Despite a promising future, phage therapy is encumbered by logistical challenges in the manufacturing process. Once a suitable phage has been identified for a particular application, it needs to be propagated using a working host, which ideally is well characterized, nonpathogenic, and free of genomically encoded prophage to prevent potential contamination of phage preparations with lysogenic phage.^5^ Such a host is often not immediately available. Next, crude phage preparations must be purified to remove endotoxin, a component of outer membranes of Gram-negative bacteria that induces septic shock.^6^ While several organic solvent extraction or affinity column purification methods exist to remove endotoxin, these approaches have widely varying efficiencies depending on the phage undergoing purification and typically result in significant loss of titer.^7^ Additionally, low shelf-stability can result in a significant loss of titer prior to clinical administration due to storage conditions, shipping conditions, and phage-dependent variability.^8−10^ Although well-defined phage products can overcome these challenges through process optimization, the lead time for personalized phage to be produced at clinically relevant titers is on the order of months.

Cell-free bacteriophage synthesis (CFBS) is a novel approach to phage production that can potentially resolve these logistical hurdles through point-of-care phage production. Cell-free expression systems (CFES) are in vitro platforms useful for synthesizing difficult-to-produce proteins, such as those that may be toxic to cells when expressed recombinantly.^11^ CFES are composed of transcription/translation machinery (TXTL) usually derived from cell extracts, an energy buffer containing nucleotides, amino acids, and reagents to support ATP recycling, and a DNA template encoding the protein of interest (Figure S1a). In some cases, mg/mL scale protein synthesis can be achieved in under 24 h relying on solely endogenous TXTL.^12^

The modular nature of cell-free systems allows for the optimization of each reaction component. The DNA template purification method (e.g., miniprep vs ethanol precipitation), structure (circular vs linear), and design (orientation of gene regulatory elements) all contribute to CFES yields and reproducibility.^13−15^ Energy buffers can be to enhance protein synthesis, primarily by adjusting magnesium (Mg^2+^) and potassium (K^+^) salt concentrations as well as by supplementation with stabilizers or enzymatic cofactors.^14,16^ The TXTL can be modified to improve yields by altering growth conditions (e.g., media composition, temperature, flask vs bioreactor) of donor strains, lysate preparation method (e.g., French-press vs sonication, runoff reactions, lysate dialysis), or genetic engineering of donor stains to improve protein/mRNA stability or transcription/translation rates.^17,18^ CFBS builds upon CFES to produce phage using purified phage genomes as templates and an energy buffer supplemented with polyethylene glycol (PEG) as a molecular crowder and dNTPs to enable phage genome replication (Figure S1b).^19^

CFBS bypasses the need to find a propagation host as TXTL is cell-free and generally extracted from a well characterized source, such as *E. coli* BL21.^20^ TXTL donor strains can be engineered to be endotoxin-free, thus avoiding endotoxin purification steps.^6,21^ Phage shelf-stability issues can potentially be resolved by lyophilization of each component of the cell-free reactions (i.e., TXTL, energy buffer, phage DNA) enabling “just-add-water” on-demand phage production at points-of-care.^21,22^ Several CFBS process improvements regarding TXTL donor growth conditions and energy buffer composition have been recently described^23,24^ for systems derived from *E. coli* BL21 Rosetta2 lysates, but there are yet to be reports of strain engineering to influence CFBS yields. As bacteria have evolved to combat phage infection, we hypothesize that even strains engineered to produce proteins at high titers are not optimized for bacteriophage expression. Genetically engineering the TXTL donor strains may also facilitate the study of the bacteriophage life cycle and host-phage interactions.

To elucidate the impact of gene function on CFBS yields, we selected coliphage T7 as a model phage to be synthesized using *E. coli* BL21 (non-Rosetta) as the TXTL donor. We choose T7 as it is perhaps the most well characterized model phage and has featured in most CFBS peer-reviewed literature.^18,23,25−27^ Previous genome-wide investigations of T7-host interactions identified at least 11 genes essential to T7 replication in vivo, but most of these are related to lipopolysaccharide (LPS) biosynthesis.^28,29^ LPS presented on the outer membrane is the receptor by which T7 recognized its hosts, without which infection cycles cannot initiate.^29^ For CFBS, phage infection is not a relevant process as phages are synthesized from naked DNA. Therefore, we interrogate the genetic basis of noncell surface host-phage interactions.

**Figure 1:**
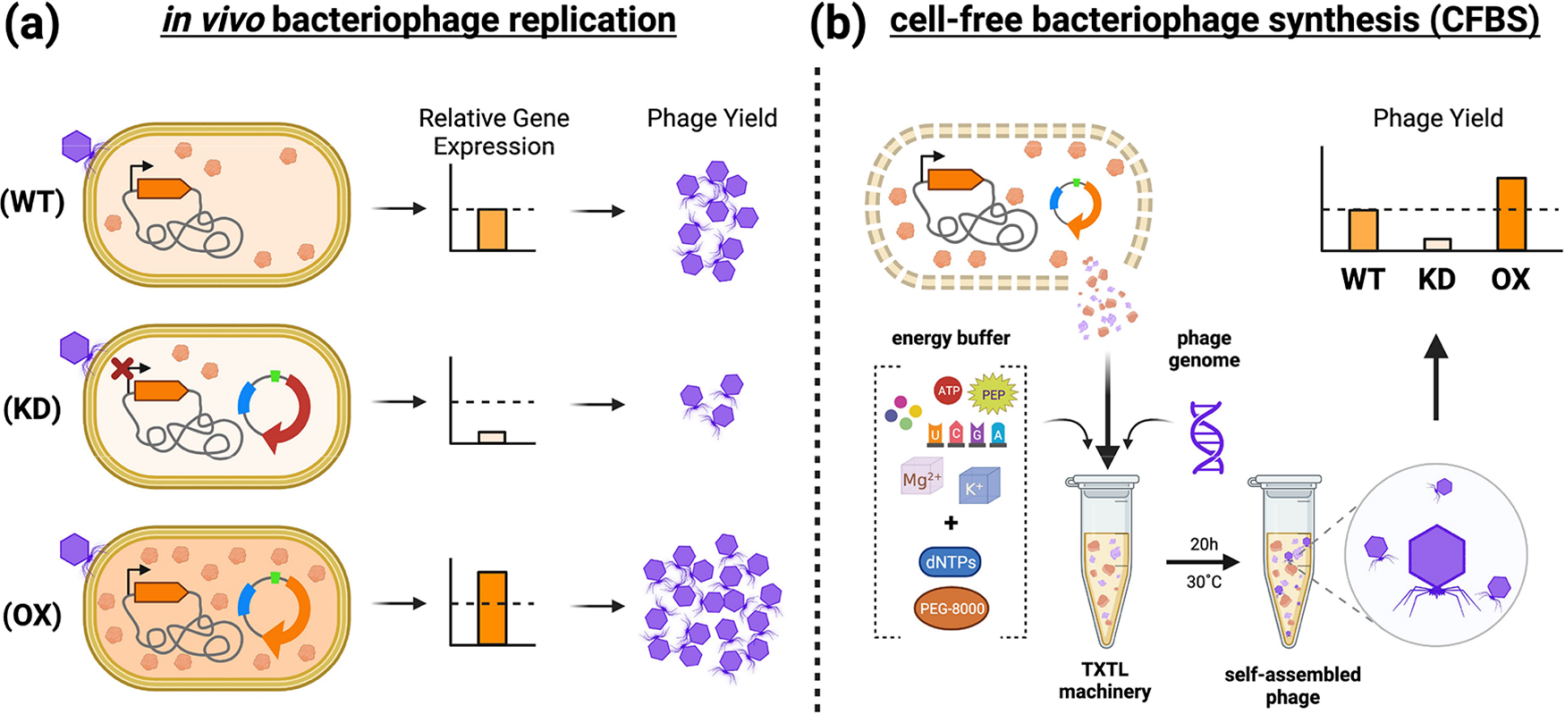
Gene expression background in the TXTL source influences CFBS yields. The number of progeny resulting from bacteriophage T7 infection of host *E. coli* BL21 is influenced by the genetic background of the host at the time of infection. Knockdown (KD) or overexpression (OX) of certain genes may have a positive, negative, or neutral impact on T7 progeny yield relative to wild-type BL21 (WT) (a). Cell-free bacteriophage synthesis (CFBS) can be modulated by modifying the genetic background of the source of transcription and translation (TXTL) machinery derived from cell lysates (b).

In this study, potential T7 CFBS effectors were interrogated by inducible CRISPR-interference (CRISPRi)-mediated gene knockdown and inducible pBAD vector overexpression. CRISPRi was chosen over chromosomal gene knockouts to allow tunable gene repression and investigating the impact of essential genes on T7.^28^ Here, we developed a facile approach for identifying phage effector genes to investigate phage-host intracellular interactions and inform strain engineering for improved CFBS yields (Figure 1).

## Results and Discussion

In this study, we examine the effect of gene expression on T7 phage fitness through the use of CRISPRi-based repression and plasmid-based overexpression. We selected 18 genes that we hypothesize will have an effect on T7 fitness.

T7 gene expression is coordinated to first inhibit host transcription and translation followed by T7 gene transcription by the T7 RNA polymerase (T7 RNAP), translation of replication and structural proteins, DNA replication, phage assembly, and lysis, which is triggered by accumulation of a critical concentration of holins.^25^ Efficient T7 replication is dependent on the utilization of host resource pools for macromolecular assembly as well as relative rates of transcription and translation.^30^

Our selected potential T7 effector genes, they represent diverse functions in macromolecular synthesis and degradation, resource pool management, energy metabolism, redox regulation, and transcription/translation regulation. Notably, many gene targets are essential to *E. coli* BL21, T7, or both (Table 1), which is why CRISPRi-interference (CRISPRi) is used to mediate gene repression as opposed gene deletions.^28^ Here, we demonstrated a single-plasmid inducible CRISPRi-mediated knockdown (KD) system featuring nuclease-deficient Cas12a from *Francisella novicida* (d*Fn*Cas12a) controlled by the rhamnose inducible promoter, pRhaB, and coexpressed crRNA under strong constitutive promoter pJ23119 (Figure S2).^31^

**Table 1:** CRISPRi/Overexpression Gene Targets

**target gene**	**category**	**function**	**essential(***E. coli***)**^a^	**essential (φT7)**^b^	**ref.**
*infC*	protein	translation initiation factor IF-3	yes		(32)
*pgk*	energy	phosphoglycerate kinase	yes		(32)
*subH* (*ssyA*)	energy	inositol monophosphatase	yes		(32)
*hemL* (*gsa*)	protein	glutamate-1-semialdehyde aminotransferase	yes		(32)
*eno*	energy/nucleic acid	enolase in glycolysis/gluconeogenesis/mRNA degradosome	yes		(32)
*mukB*	protein/cell-division	degradosome/chromosomal DNA condensation	yes		(32)
*lexA*	transcription factor	SOS response regulator	yes		(32)
*rne*	nucleic acid	RNase E	yes	no	(29,32,33)
*nusG*	transcription factor	transcription termination factor	yes	no	(32,34,35)
*trxA*	redox regulator	oxidized thioredoxin	no	yes	(29,32)
*T7gp3 (T7 gene)*	nucleic acid	φT7 DNA replication	no	yes	(36)
*dgt*	energy	deoxyguanosinetriphosphate (dGTP) triphosphohydrolase	no	no	(29,32)
*udk*	nucleic acid	uridine kinase/cytidine kinase	no	no	(29,32)
*recC*	nucleic acid	Exodeoxyribonuclease V (RecBCD) subunit	no	no	(29,32,37)
*cyaR* (*ryeE*)	transcription factor	CyaR small RNA, oxidative stress regulator	no		(38)
*rna*	nucleic acid	Rnase I, cleaves phosphodiester bond between any two nucleotides	no		(32)
*oxyS*	transcription factor	OxyS small RNA, oxidative stress regulator	no	no	(39)
*trxB*	redox regulator	thioredoxin reductase	no	no	(29,32,40)

a Chromosomal deletion results in nonviable or nonreplicative cells.

b Host/phage chromosomal deletion causes nonproductive phage infections.

### CRISPRi-Mediated *trxA* Knockdown Influences T7 Fitness

**Figure 2:**
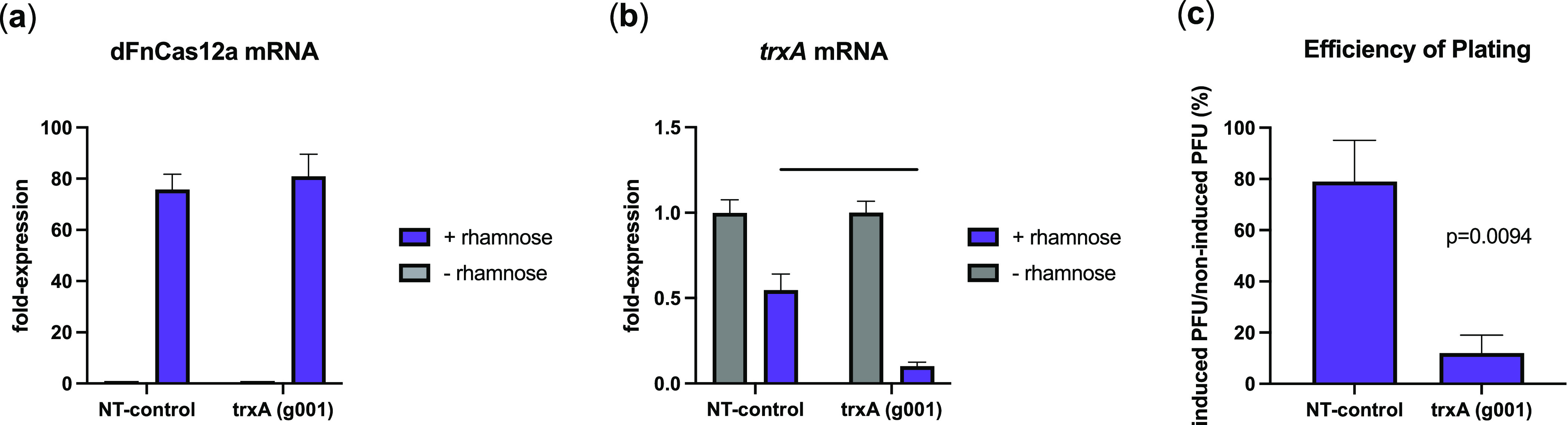
Inducible CRISPRi of *trxA* knockdown mRNA and T7 efficiency of plating (EOP). Induced expression of dFnCas12a (a) with crRNA targeting the trxA promoter results in repression of *trxA* by 90 ± 2.4% (b), which is associated with a significant efficiency of plating (EOP) reduction to 12 ± 7.0% (c). Data represented as mean ± SD (*n* = 3). Rhamnose induction significantly lowers *trxA* expression vs the NT-control (*p* < 0.0001, 2-way ANOVA). Welch’s two-tailed *t* test was performed indicating significant reduction of EOP (*p* < 0.05). *p* ≤ 0.05, *; *p* ≤ 0.01, **; *p* ≤ 0.001, ***.

To demonstrate the efficacy of this system, a CRISPRi vector with a crRNA targeting the *trxA* promoter (pCRJ001_g001, inserted via Golden Gate assembly) was transformed into T7 host *E. coli* BL21.^41^ The *trxA* gene encodes thioredoxin 1, a nonessential *E. coli* protein involved with cytoplasmic redox homeostasis and an essential host-factor for T7 genome replication.^32,42^ In *trxA-*deficient mutants, T7 will still induce host lysis and produce progeny virions, but will not replicate, making *trxA* an ideal target to demonstrate the effects of gene knockdown on T7 fitness.^43^ The functionality of the CRISPRi system was evaluated using RT-qPCR and direct plaque assays to enumerate T7 progeny resulting from the infection of host *E. coli* BL21 with a *trxA* knockdown (*trxA*-KD) background. Gene repression was induced in log-phase cells with 2% L-rhamnose (w/v) for 4 h, at which point, cells were harvested for RNA extraction and T7 infection assays. RT-qPCR revealed 90 ± 2.4% (SD, *n* = 3) drop in *trxA* mRNA compared to the noninduced control carrying the same CRISPRi vector (Figure 2b). Rhamnose induction increased d*Fn*Cas12a mRNA by over 70-fold compared to uninduced controls (Figure 2a). T7 infection in a *trxA*-KD background resulted in an efficiency of plating (EOP) of 12 ± 7% relative to the uninduced control (Figure 2c). The *trxA-*KD EOP was also significantly lower compared to that of the nontargeting (NT) control strain (vector pCRJ001_g037) (*p* < 0.01, Welch’s two-tailed *t* test).

### CRISPRi Screen of Potential T7 Fitness Effector Genes

Having confirmed that our inducible CRISPRi system could be used to influence T7 fitness, additional CRISPRi vectors were prepared to target each of the potential T7 effectors selected in this study (Table 1). Two vectors were designed for each gene with one crRNA directed toward promoters and one toward coding sequences (CDS) as near to transcription start sites (TSS) as possible as available PAM sites allowed. The exception was T7 exonuclease gene 3 (*T7gp3*), the only T7 gene targeted in this experiment for which only the CDS was targeted. Each CRISPRi vector was then transformed into BL21 for experiments evaluating the impact of gene knockdowns on T7 fitness.

**Figure 3:**
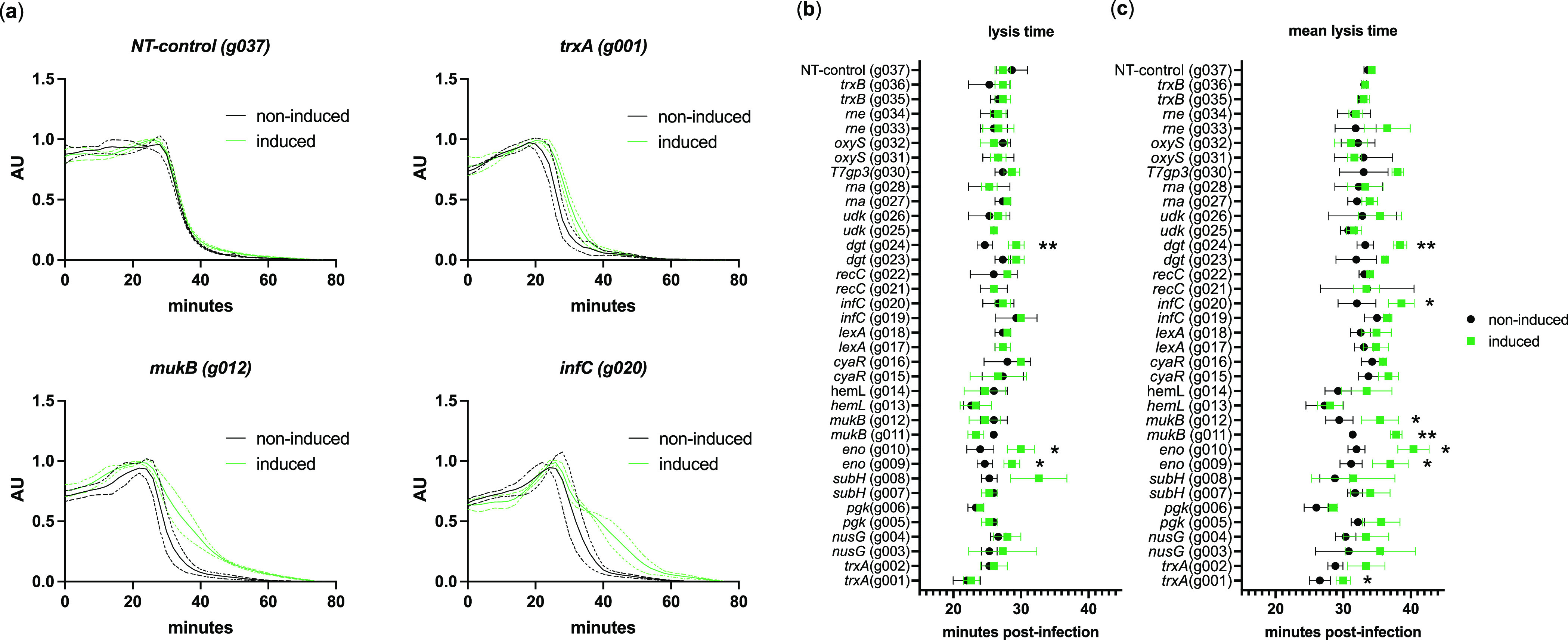
CRISPRi-mediated gene repression in *E. coli* BL21 has varied impact on T7 lysis onset time and mean lysis time (T50%). CRISPRi induced by 2% (w/v) L-rhamnose for 4 h changes lysis profiles of T7 infecting log-phase *E. coli* BL21 depending on gene target and whether crRNA targets promoters (odd numbers) or coding sequences (even numbers). A nontargeting control was included in each experiment (g037). Optical densities (OD) were normalized with a max OD = 1 for clearer comparisons. Shaded regions represent the max and min OD of triplicate experiments performed on different days. Representative curve effects (a): neutral (NT-control), shift (*trxA* (g001)), widening (*mukB* (g012)), and shoulder (*infC* (g020)). Lysis onset time (b) and mean lysis time (c) are represented as mean ± SD (*n* = 3). Welch’s two-tailed *t* test was performed indicating significant change in lysis and mean lysis timing for as a result of CRISPRi induction (*p* < 0.05). *p* ≤ 0.05, *; *p* ≤ 0.01, **.

Lysis time courses and EOP assays were used to compare the relative T7 fitness. Here, lysis onset time is defined as the time post-T7 infection at which optical density begins to decrease as previously described.^25^ Log-phase BL21 carrying CRISPRi vectors (OD = 0.3) were infected with T7 to a multiplicity of infection (MOI) = 3 and then OD kinetics were tracked (Figures 3a and S2). Overall, in cultures without CRISPRi induction, the lysis onset was ∼25 min, later than the typical T7 lysis onset of ∼15 min.^25^ We attribute this to the metabolic burden of plasmid maintenance and chloramphenicol in selective media. Initial screens found delayed lysis onset in induced CRISPRi strains with crRNAs targeting the *dgt* CDS (g024) (*p* < 0.01) and both the *eno* promoter (g009) and CDS (g010) (*p* < 0.05) relative to the same strains without CRISPRi induction Figure 3b.

While only three CRISPRi targets resulted in significant changes in lysis onset time, examination of lysis curves revealed an extended lysis period for many targeted genes, as indicated by the differences in the lysis kinetic profiles when CRISPRi was induced (Figure 3a). Based on this observation, we also compared mean lysis times, which represents the time at which 50% of cells are lysed.^44^ In addition to *dgt* and *eno*, repressors *trxA* promoter (g001), *mukB* promoter and CDS (g011 and g012 respectively), and *infC* CDS (g020) were also found to have significant delays in mean lysis time. This suggests that each of these effector candidates may warrant further investigation.

**Figure 4:**
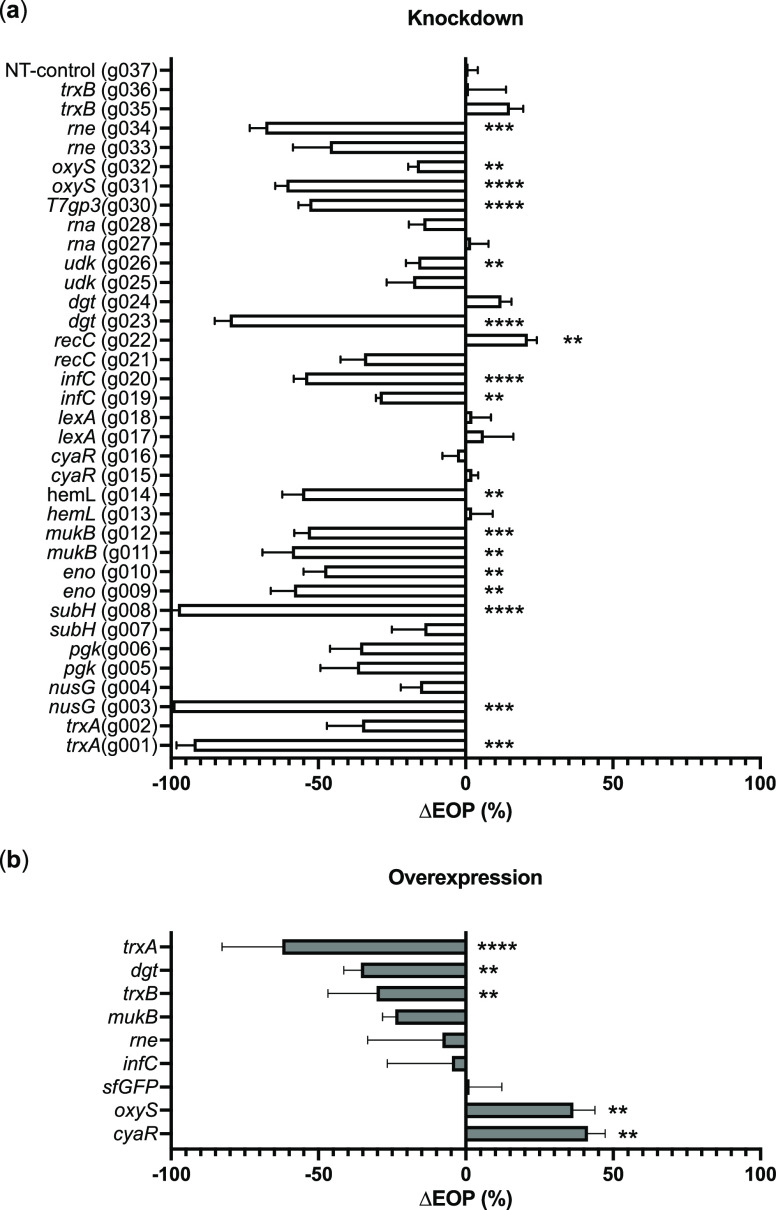
Gene overexpression and CRISPRi-mediated gene repression modulates efficiency of plating (EOP). Log-phase *E. coli* BL21 knockdown (a) and overexpression (b) strains were infected with phage T7 at MOI = 0.0001. After 30 min (roughly one lysis cycle) at 37 °C, further phage replication was stopped by adding chloroform to each culture followed by pipet mixing. Phage yields were enumerated by plaque counting on double-layer agar plates containing *E. coli* BL21 as a propagation host. Efficiency of plating (EOP) represents the fraction change in assembled phage between gene-repressed CRISPRi strains and their nonrepressed counterparts carrying the same plasmid without CRISPRi induction (a) or fraction change in assembled phage between overexpressing strains and their noninduced counterparts carrying the same plasmid (b). Data represented as mean ± SD (*n* = 3). Welch’s two-tailed *t* test was performed indicating significant change in EOP as a result of CRISPRi induction (*p* < 0.05). *p* ≤ 0.05, *; *p* ≤ 0.01, **; *p* ≤ 0.001, ***; *p* ≤ 0.0001, ****.

While indicative of altered T7 life cycle kinetics, lysis onset and mean lysis timing are not necessarily indicative of effects on the number of progeny phage resulting from infection.^27^ Therefore, the efficiency of plating (EOP) in knockdown strains was also evaluated. Log-phase CRISPRi *E. coli* BL21 stains (OD = 0.3) were infected at MOI = 0.0001 (1 virion per 10,000 cells) for 30 min, and then, phage replication was halted with chloroform and progeny phage enumerated by plaque assay using wild-type BL21 as the host. EOP assays found that most CRISPRi constructs had a significant negative impact on T7 titer, with the exceptions of those targeting *lexA, trxB, rna, pgk, cyaR*, and *recC* (Figure 4a). SOS response transcriptional regulator *lexA* expression is tightly limited under normal growth conditions, so its repression was not expected to elicit a strong effect on T7 fitness.^45^ Likewise, starvation response regulator *cyaR* repression did not affect the T7 lysis. RNase I encoded by *rna* localizes in the periplasm and is thus unlikely to interfere with T7 mRNA stability.^46^ Phosphoglycerate kinase (*pgk*) did not affect lysis timing or EOP, which is unexpected given the lysis delay and EOP drop induced by the enolase (*eno*) knockdown as both enzymes are part of the canonical glycolysis pathway.^47^

*eno* was chosen as a CRISPRi target because of its roles in glycolysis, as part of the mRNA degradosome complex, and interactions between enolase and other phage. Notably, enolase associates with RNase E (*rne*), whose major role is degradation of mRNA. The T7 early gene gp0.7 inhibits RNase E activity early during infections to stabilize phage-derived mRNAs.^33^ There is evidence of protein–protein interactions between coliphage T1 and *E. coli* enolase and direct inhibition of enolase by *Bacillus subtilis* phage SPO1, so it stands to reason that there may be interactions between T7 and enolase.^33,48,49^ Because *rne* inhibition is part of the T7 lifecycle, we expected *rne* knockdown to have a neutral to positive effect on EOP. However, as with *eno, rne* knockdown lowered EOP. This was perhaps due to the impairment of RNase E's role in rRNA maturation, which could lead to reduced translational capacity. In silico modeling of infection kinetics suggests that the translation rate is the primary bottleneck in T7 progeny assembly.^30^ The hypothesis that translation rates influence T7 fitness is supported by our observations of lysis delay and EOP loss in translation initiation factor IF-3 (*infC*) (Figure 4a).

Of the essential *E. coli* genes investigated, *mukB, subH, hemL,* and *nusG* repression caused a significant loss of EOP (Figure 4a). While each of these knockdown strains were viable under experimental conditions, they also experienced severe growth rate defects, which have been associated with diminished phage bursts.^30^

Knockdown of known antagonistic *E. coli* genes *udk* (uridine-cytidine kinase) and *dgt* unexpectedly resulted in a lower EOP (Figure 4a). T7 encodes Udk and Dgt inhibitors (gp0.7 and gp1.2, respectively), so we hypothesized that repressing these genes could increase T7 fitness. However, the interfering with *udk* only caused a small nonsignificant drop in EOP. The *dgt* promoter repressor (dgt023) caused a significantly lower EOP, perhaps due to perturbed ribo- and deoxyribonucleotide pool homeostasis, thereby preventing normal T7 activities.^29^

CRISPRi-based knockdown of the small regulatory RNA *oxyS* also decreased EOP (Figure 4a), which was consistent with our expectation. OxyS acts as a regulator of stress-response sigma factor RpoS (σ^S^) expression and oxidative stress response.^50^ Previous studies demonstrate that Δ*oxySE. coli* has higher expression of RpoS and RpoS-regulated genes relative to wild-type and that inducible *oxyS* expression results in RpoS suppression.^50^ Early T7 transcription is mediated primarily by *E. coli* RNA polymerase-sigma 70 complex (Eσ^70^) but can also be carried out using the alternate Eσ^S^ complex.^51^ During middle and late gene transcription, Eσ^70^ and Eσ^S^ are inhibited by T7 gp2 and gp5.7, respectively, and transcription is taken over by T7 RNAP. T7 gp2 mutants experience abortive infections associated with “aberrant” transcription (interrupted transcripts and atypical, terminator read-through) caused by competition between Eσ^70^ and T7 RNAP.^52^ We suspect that the loss of EOP in *oxyS*-knockdown strains may be due to incomplete Eσ^S^ inactivation due to greater background RpoS accumulation and competition between active Eσ^S^ and T7 RNAP.

Another interesting observation was the neutral impact of *trxB* (thioredoxin reductase) knockdown on EOP. In the TrxA/TrxB redox system, TrxA acts as a recyclable reducing agent with diverse roles in over 80 protein–protein interactions and transcriptional regulation of at least 26 genes.^53^ These complex interactions are implicated in most cellular processes including oxidative stress response, translation, and energy transduction. TrxB recharges oxidized TrxA to restore the pool of reduced-form TrxA. Under normal conditions, balanced TrxA concentrations and TrxB activity maintain the majority of TrxA in its reduced form.^54,55^ TrxA exists in its reduced state in the functional T7 DNA polymerase holoenzyme and does not meaningfully interact with T7 DNA polymerase in its oxidized state.^40^*trxB* deletion causes cytosolic TrxA to exist solely in its oxidized form, which has been shown to lower EOP by 10^–8^-fold for coliphage f1, which also requires reduced form TrxA.^40,56^ As discussed above, *trxA* knockdown lowered the EOP, likely by depletion of free TrxA (Figures 2 and 4a). We anticipated that *trxB* knockdown would cause a similar effect by depleting reduced-form TrxA. The lack of impact on EOP in the *trxB-*KD strains suggests that there is sufficient TrxB activity to supply adequate reduced-form TrxA to support T7 replication.

The *recC* CDS (g022) was the only target whose knockdown resulted in a significant positive effect on EOP (+21 ± 3.1%). RecC was selected as a target because of its role its role in the double-stranded DNA repair RecBCD holoenzyme, which has exonuclease activity on linear DNA such as the T7 genome.^57^ Deletion of *recB* or *recC* results in loss of nuclease activity.^37^ Exonuclease inhibitor GamS is typically included in CFES to protect linear DNA templates from RecBCD. Improved EOP here was consistent with recent work by Batista et al. showing that TXTL with *recB* or *recBCD* deletions improved CFES sfGFP yields for linear templates compared to wild-type TXTL.^14^

Inducer titration experiments were conducted on a subset of CRISPRi strains to determine if gene knockdown effects were tunable (Figure S4a). The *trxA* knockdowns showed concentration dependence, where higher L-rhamnose concentrations caused greater loss of EOP. Repression of *rna* had no impact on EOP at any concentration of rhamnose, consistent with our previous results (Figure 4a). At lower rhamnose concentrations (0.02–0.1% w/v), *recC* repression lowered the EOP in contrast to the EOP gains observed at higher concentrations (0.2–2% w/v).

In brief, we demonstrated that a novel d*Fn*Cas12a-based single-plasmid CRISPRi system could be used to influence phage fitness in an inducible and tunable manner. The system can be used to knockdown essential and nonessential genes of host and phage as well as genes encoding sRNAs rather than proteins. The strength of interference with phage fitness depends on the strength of gene repression, so care must be taken when designing crRNAs as which provides tighter control, targeting promoters versus CDS, appears to be gene-dependent. One approach could be to design additional crRNA constructs for each gene or to multiplex promoter and CDS targeting as our *Fn*Cas12a system is readily multiplexed by constructing vectors with consecutive guide RNA scaffold arrays.^58^

### Overexpression of Potential Phage Fitness Effectors

To complement the investigation of phage effectors by CRISPRi, we sought to explore modulating T7 fitness by effector overexpression. l-Arabinose-inducible pBAD vectors were constructed to overexpress a subset of the T7 effector targets. This subset was chosen from among the genes whose repression caused significant loss in EOP (Figure 4a) under the hypothesis that if repression causes a negative impact on T7 fitness, overexpression may have a positive impact. Among these genes, only overexpression of sRNAs *oxyS* and *cyaR* increased T7 EOP (*p* < 0.05 Welch’s two-tailed *t* test), *dgt, trxB, and trxA* decreased EOP, and *infC* and *rne* had a neutral effect as did the control of overexpressed sfGFP (Figure 4b). *mukB* overexpression, which has also been implicated in acetate tolerance,^59^ also decreased EOP but was not significant. In contrast to the knockdown studies, none of these overexpressions impacted lysis timing or the profile of their lysis curves.

We suspect that CyaR and OxyS may support T7 transcription by interfering with RpoS translation.^38,50^ OxyS may also act to reduce oxidative stress on T7 by activating oxidative stress-response in an RpoS-independent manner.^60,61^

Huber et al. found that a Dgt overexpressing *optA1* mutant *E. coli* maintained 50× higher dGTPase concentrations, which lowered dGTP pools 5-fold.^62^ Wild-type T7 is able to replicate in these dGTP depleted backgrounds, but foundational studies utilizing *optA1* strains do not report impact on EOP.^62−64^ Here, we again demonstrate that T7 does propagate in a Dgt overexpressing strain but EOP is diminished.

Unexpectedly, TrxA and TrxB overexpression decreased the EOP. We expected these genes to have a positive effect on T7 titer in contrast to the near abolition of T7 replication in *trxA* CRISPRi strain g001. It is unclear why this occurred, but overexpression of these genes may have interfered with redox balance or had other unpredictable effects, given TrxA’s many protein–protein and protein–DNA interactions.^53,55^

As with the CRISPRi constructs, our overexpression strains showed an l-arabinose concentration dependence of EOP effects (Figure S4b). Most notably, there is a negative relationship between the l-arabinose concentration (0.002–0.2% w/v) and EOP in the pBAD-*trxA* strain. pBAD-*oxyS* and pBAD-*cyaR* only reach a significantly increased EOP at 0.2% l-arabinose. Likewise, pBAD-*dgt* only significantly lowers EOP at 0.2% l-arabinose. Meanwhile, pBAD-*infC* showed a steady but small EOP increase up to 0.02% l-arabinose but dropped back to the baseline at 0.2%. Importantly, T7 EOP was insensitive to induction of control pBAD-sfGFP at all l-arabinose concentrations.

### Effect of Gene Expression Background in the TXTL Source on Cell-Free Expression Systems and Bacteriophage Synthesis

To determine if in vivo effectors of T7 fitness had the same qualitative effect in cell-free systems, CRISPRi and overexpression strains were selected to prepare transcription/translation machinery (TXTL) for cell-free protein express systems (CFES) and cell-free bacteriophage synthesis (CFBS) based on prior research, indicating their usefulness in cell-free protein synthesis or positive effects on T7 fitness during in vivo studies. Exogenous IF-3 (*infC*) supplementation, *recC* deletion, and *rna* deletion each have been shown to improve CFES yields of GFP.^14,46,65^*oxyS*- and *cyaR*-overexpression strains were chosen as positive in vivo effectors, *dgt*-overexpression, and *trxA-*knockdown strains as likely negative effectors and *trxA*-overexpression for its strong negative effects. All lysates were harvested 4 h after induction with an appropriate inducer and harvested in mid log-phase with OD_600_ ∼ 2 to 3 yielding total protein yields ranging from 15 to 25 mg/mL. No growth defects were observed, but *recC-*KD strains had an extended lag-phase (∼1 h) before entering log-phase. Lysates were produced from each strain in duplicate grown from different colonies.

**Figure 5:**
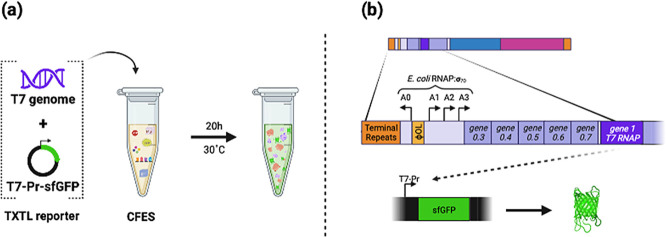
T7 genome pJl1-T7-Pr-sfGFP cascade phage-dependent TXTL mechanism. A cell-free expression system synthesizing sfGFP was used to troubleshoot CFBS reaction. 0.5 nM T7 genome was included in a standard CFES reaction using sfGFP expression controlled by T7 RNA polymerase (RNAP) (a). Transcription of sfGFP occurs only if T7 RNAP (T7 gene 1) itself is transcribed by endogenous *E. coli* RNAP (b). sfGFP expression reflects functional endogenous TXTL capacity and T7 RNAP synthesis and transcription activity.

**Figure 6:**
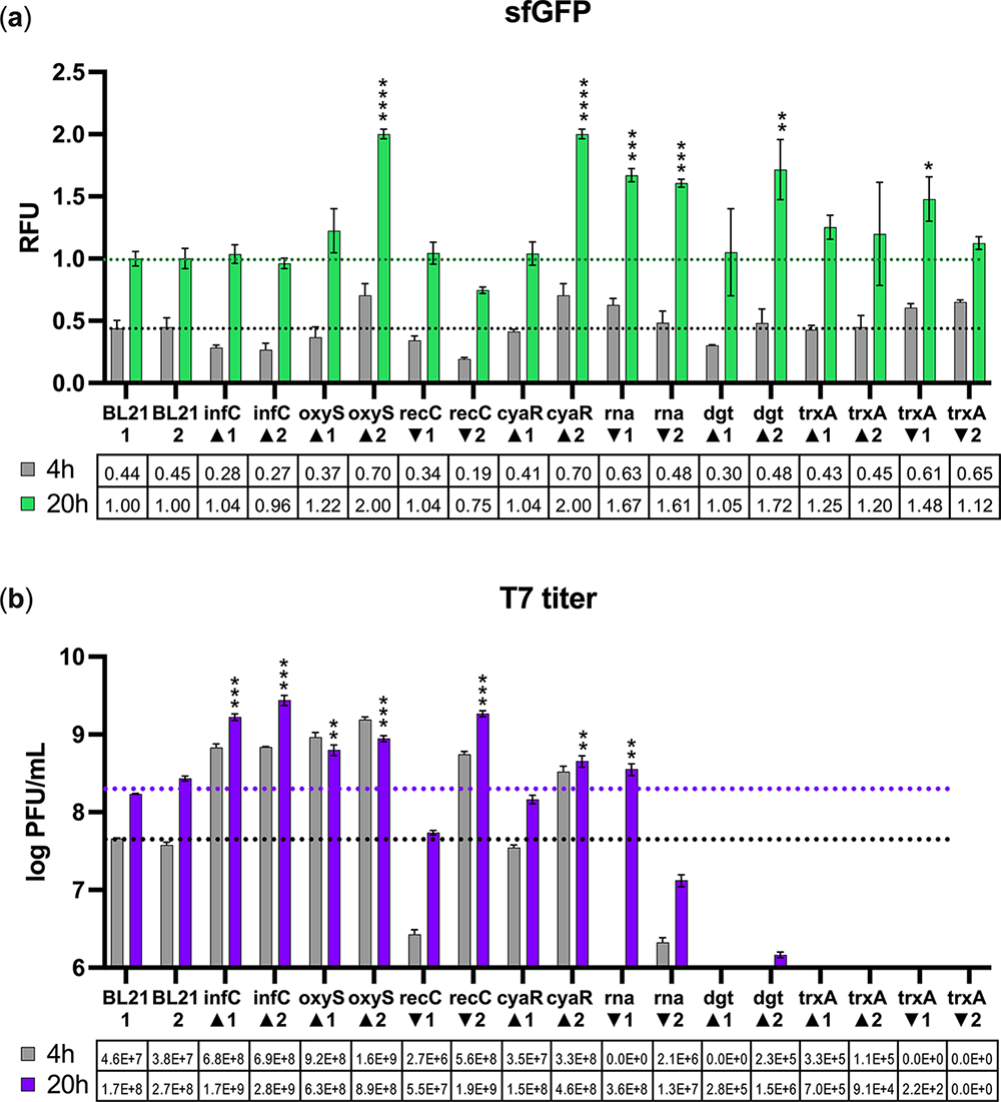
Cell-free protein and bacteriophage synthesis yields are influenced by genetic background of the TXTL donor. Biological duplicates (1 and 2) of cell lysates were prepared from *E. coli* BL21 overexpression (UP ▲) or repression (DOWN ▼) of various effector genes. Protein synthesis (a) was carried out using the T7 genome pJl1-T7-Pr-sfGFP cascade (Figure 5). CFBS yields (b) were measured by plaque count assays using wild-type BL21 as a propagation host. Gray bars indicate protein and phage yields at 4 h. Black dotted lines indicate 4 h yields in wild-type BL21 lysates and colored lines indicate 20 h yields. Data show mean ± SD (*n* = 3). Welch’s two-tailed *t* test was performed, indicating significant increase in sfGFP fluoresce or T7 titer relative to the wild-type *E. coli* BL21 baseline (*p* < 0.05). *p* ≤ 0.05, *; *p* ≤ 0.01, **; *p* ≤ 0.001, ***; *p* ≤ 0.0001, ****.

To confirm that each lysate could carry out transcription from the T7 genome and produce proteins, we developed a simple cascade CFES reaction containing T7 genomes and the fluorescent reporter plasmid pJL1-T7-Pr-sfGFP (Figure 5a). In these reactions, endogenous *E. coli* RNA polymerase transcribes T7 early genes including T7 RNAP (*gp1*) (Figure 5b). T7 RNAP then drives sfGFP expression via the reporter plasmid. sfGFP is synthesized only in the presence of functional *E. coli* TXTL machinery and T7 transcriptional activity. CFES using each lysate showed sfGFP yields matching (*infC*_UP, *recC*_DOWN, *trxA*_UP) or exceeding that of wild-type BL21 TXTL after 20 h (*oxyS*_UP, *cyaR*_UP, *rna*_DOWN, *dgt*_UP) (Figure 6a). However, these CFES reactions did not result in detectible plaque forming units (PFU) due to the lack of dNTPs supplied.

Having confirmed that all lysates were capable of T7-mediated protein synthesis, CFBS reactions were carried out. *infC*_UP, *oxyS*_UP, *cyaR*_UP, *recC*_DOWN, and *rna*_DOWN all matched or exceeded wild-type BL21 lysate T7 yields by 20 h for at least one biological replicate with *infC*_UP lysates resulting in the greatest fold increase in T7 titer (9.7–16-fold) (Figure 6b). Consistent with expectations, *dgt*_UP and *trxA*_DOWN eliminated T7 yields in some reactions and lowered yields by 10^2^–10^5^-fold in reactions where T7 was detected. *trxA*_UP reactions also had T7 yields diminished by ∼10^3^-fold, which was consistent with in vivo effector assays. The absolute values of the phage yield in this study are comparable with previous efforts; the unmodified BL21 strain’s lysate produced over 10^8^ PFU/mL after 20 h is on par with results from similar conditions after 12 h.^26^ Of note, BL21 was chosen as the lysate donor in this study rather than BL21 Rosetta2 or BL21(DE3) Rosetta2 typical in other cell-free expression systems. This was done to ensure that the T7-host and T7-CFBS interactions more closely reflect natural phage-host interactions.

It is notable that high or low CFES sfGFP yields did not necessarily predict CFBS T7 yields (Figure 6). For instance, *infC*_UP matched wild-type BL21 TXTL for sfGFP yields but increased T7 yields, whereas *dgt*_UP and *trxA*_DOWN exceeded wild-type BL21 for sfGFP yields but suffered substantial loss of T7 titer. This suggests that the influence of TXTL background on phage yields is not strictly tied to protein synthesis capacity. Multiple reports describe differential optimization of cell-free synthesis of different proteins usually by varying reaction conditions (e.g., temperature, volume), composition (e.g., [Mg^2+^], linear vs circular template, redox state), and bacterial source of transcriptional/translation machinery (e.g., *E. coli* A19 vs BL21 vs non-*E. coli* bacteria).^14,66−68,69^ These factors affect the physicochemical environment in which protein synthesis takes place, and some degree of optimization may be required for each new protein or phage to be produced using cell-free systems. On the road to engineering of chassis strains for CFBS TXTL donors, further investigation is required to determine if the positive/negative effectors described here are unique to T7 or can be generalized to develop a generalized CFBS platform optimized for more production of more diverse phage.

The trends for effector impact on T7 fitness were the same in vivo and in CFBS, (e.g., positive effector in vivo and in CFBS), suggesting in vivo screening of effectors is an appropriate approach for determining TXTL backgrounds that may support CFBS yield improvements.

Prior work has demonstrated that CFES supplementation with exogenous TrxA and IF-3 improved synthesis of sfGFP transcribed from plasmids by T7 RNAP.^65^ Here, the overexpression of IF-3 increased T7 but did not have a significant impact on sfGFP yields. Conversely, TrxA overexpression negatively impacted CFBS T7 titers again without a significant impact on sfGFP. These results may be explained by the impact of pleiotropic effects of overexpressed endogenous protein. IF-3 does not have any known transcriptional regulation roles, whereas TrxA interacts with numerous proteins and genes with a wide variety of metabolic functions and indirectly as a transcriptional regulator.^53^ Likewise, OxyS and CyaR primarily function as transcription and transcription regulators of *E. coli* genes and may significantly alter the proteome of TXTL machinery. To avoid pleiotropic effects, future work supplementing CFBS with exogenous proteins could help elucidate whether T7 effectors influence CFES yields directly or indirectly. In particular, proteomics of *oxyS*- and *cyaR-*OX lysates may reveal more CRISPRi and OX targets to improve CFBS yields while minimizing global transcriptional changes. A combination of proteomics, CRISPRi, and OX has the potential to inform the design of mutant *E. coli* lysates optimized for complex phage production rather than simply protein synthesis.

## Conclusions

We demonstrate here the rational engineering of *E. coli* BL21 for improved T7 phage production from both standard infection and a cell free bacteriophage synthesis platform (CFBS). We show that using a d*Fn*Cas12a-based CRISPRi knockdown method and plasmid-based overexpression, most alterations to the host transcriptome are deleterious to phage production in vivo. This comports with the idea that phage replication is already mostly optimized within its host strain. We show that improved protein production correlates to but does not fully predict improved phage production from CFBS. However, we observed that changes to host expression that lead to increased protein production and reduced RNA degradation lead to greatly increased phage production in CFBS. Translation initiation factor-3 (*infC*) overexpression and *recC* repression have both been demonstrated as positive modifications to protein cell-free expression systems, particularly in systems with linear DNA templates in the case of *recC* repression. Additionally, regulatory small RNAs OxyS and CyaR increased CFBS yields. It may be that these sRNAs are interfering with RpoS expression, but further studies are warranted to determine the mechanism of improvement. Combinatorial overexpressions and repressions may further improve the phage production, but interactive effects, especially for the regulatory small RNAs, may prove complex.

Cell-free synthesis of bacteriophages is a powerful platform for the design and construction of engineered bacteriophages for biotechnology and therapeutic purposes. We envision that such a platform may be able to rapidly synthesize multiple bacteriophages to yield a “phage cocktail” that is more difficult to evolve resistance to compare to single bacteriophage. The primary benefit of CFBS is that it does not require a successful cell surface-tail fiber interaction to yield viable phage, making engineering phages that do not infect the lysate’s donor strain possible. This potentially expands the range of phages that can be engineered, especially those that are difficult to transform with whole phage genomes. The key question remains on how phylogenetically far the CFBS lysate donor strain can be from the phage’s native host and still yield viable phage virions at high titers and the underlying genetic mechanisms that limit this range. In time, nonmodel bacteria-based CFBS platforms may be generated for the generation of bacteriophage targeting the most notorious multidrug resistant pathogens.

## Materials and Methods

### Bacteria and Bacteriophage Strains and Growth Conditions

*E. coliDH5*α [genotype F^–^Φ80*lac*ZΔM15Δ(*lac*ZYA-*arg*F) U169 *rec*A1 *end*A1 *hsd*R17(r_K_^–^, m_K_^+^) *pho*A *sup*E44 *thi*-1 *gyr*A96 *rel*A1 λ-] was used for plasmid cloning (NEB, Ipswich, MA). *E. coli* BL21 ATCC BAA-1025 [genotype F^–^*ompT lon hsdS*_B_(r_B_^–^ m_B_^–^) *gal dcm* [*malB*^*+*^]_K-12_(λ^S^)] was used for phage propagation, in vivo phage fitness and quantification assays, and as a source of cell extract for cell-free bacteriophage synthesis (CFBS) (American Tissue Culture Collection, Manassas, VA). *E. coli* cells were grown at 37 °C in Luria–Bertani (LB) medium (10 g/L tryptone, 5 g/L yeast extract, 10 g/L sodium chloride in Milli-Q water) supplemented with antibiotics as appropriate (ampicillin, 100 μg/mL; chloramphenicol, 50 μg/mL; kanamycin, 35 μg/mL). For T7 phage experiments, φLB (LB with 2.5 mM MgSO_4_ and 2.5 mM CaCl_2_) was used. Solid media incorporated 1.5% agarose. Liquid culture was performed at 37 °C and agitated at 250 rpm unless otherwise noted. All strains and plasmids used in this study are listed in Table 2 and 3, respectively. Primers and DNA oligos were purchased from Integrated DNA Technologies (Coralville, IA) and are listed in (Table S1).

**Table 2:** *E. coli* and Phage Strains Used in This Study

**strain**	**relevant genotype**	**source**
E. coli DH5α	F^–^ Φ80*lac*ZΔM15Δ(*lac*ZYA-*arg*F) U169 *rec*A1 *end*A1 *hsd*R17(r_K_^–^, m_K_^+^) *pho*A *sup*E44 *thi*-1 *gyr*A96 *rel*A1 λ-	NEB C2987I
E. coli BL21	F^–^*ompT lon hsdS*_B_(r_B_^–^ m_B_^–^) *gal dcm* [*malB*^*+*^]_K-12_(λ^S^)	ATCC BAA-1025
*E. coli* bacteriophage T7	N/a	ATCC BAA-1025-B2

**Table 3:** Plasmids Used in This Study^a^

**plasmid**	**relevant features**	**reference or source**
pJL1-sfGFP	T7 promoter*-*T7 gene 10 RBS-sfGFP-T7 terminator *kanR* (kanamycin resistance) ColE1 ori	Addgene # 69496
pBAD-sfGFP	araBAD promoter-T7gene 10 RBS-sfGFP-rrnB1 T1 terminator *ampR* (ampicillin resistance) ColE1 ori	Addgene #54519
pCRJ001	rhaB promoter-RBS-dFnCas12a (aka dFnCpf1)-rrnB1 terminator; J23119 promoter-FnCas12a sgRNA scaffold-LacZα *cat* (chloramphenicol resistance) p15A ori	Derived from pJRJ001^77^
pCRJ001-g001	pCRJ001 derivative expressing sgRNA targeting *trxA* promoter	this study
pCRJ001-g002	pCRJ001 derivative expressing sgRNA targeting *trxA* CDS	this study
pCRJ001-g003	pCRJ001 derivative expressing sgRNA targeting *nusG* promoter	this study
pCRJ001-g004	pCRJ001 derivative expressing sgRNA targeting *nusG* CDS	this study
pCRJ001-g005	pCRJ001 derivative expressing sgRNA targeting *pgk* promoter	this study
pCRJ001-g006	pCRJ001 derivative expressing sgRNA targeting *pgk* CDS	this study
pCRJ001-g007	pCRJ001 derivative expressing sgRNA targeting *subH* promoter	this study
pCRJ001-g008	pCRJ001 derivative expressing sgRNA targeting *subH* CDS	this study
pCRJ001-g009	pCRJ001 derivative expressing sgRNA targeting *eno* promoter	this study
pCRJ001-g010	pCRJ001 derivative expressing sgRNA targeting *eno* CDS	this study
pCRJ001-g011	pCRJ001 derivative expressing sgRNA targeting *mukB* promoter	this study
pCRJ001-g012	pCRJ001 derivative expressing sgRNA targeting *mukB* CDS	this study
pCRJ001-g013	pCRJ001 derivative expressing sgRNA targeting *hemL* promoter	this study
pCRJ001-g014	pCRJ001 derivative expressing sgRNA targeting *hemL* CDS	this study
pCRJ001-g015	pCRJ001 derivative expressing sgRNA targeting *cyaR* promoter	this study
pCRJ001-g016	pCRJ001 derivative expressing sgRNA targeting *cyaR* CDS	this study
pCRJ001-g017	pCRJ001 derivative expressing sgRNA targeting *lexA* promoter	this study
pCRJ001-g018	pCRJ001 derivative expressing sgRNA targeting *lexA* CDS	this study
pCRJ001-g019	pCRJ001 derivative expressing sgRNA targeting *infC* promoter	this study
pCRJ001-g020	pCRJ001 derivative expressing sgRNA targeting *infC* CDS	this study
pCRJ001-g021	pCRJ001 derivative expressing sgRNA targeting *recC* promoter	this study
pCRJ001-g022	pCRJ001 derivative expressing sgRNA targeting *recC* CDS	this study
pCRJ001-g023	pCRJ001 derivative expressing sgRNA targeting *dgt* promoter	this study
pCRJ001-g024	pCRJ001 derivative expressing sgRNA targeting *dgt* CDS	this study
pCRJ001-g025	pCRJ001 derivative expressing sgRNA targeting *udk* promoter	this study
pCRJ001-g026	pCRJ001 derivative expressing sgRNA targeting *udk* CDS	this study
pCRJ001-g027	pCRJ001 derivative expressing sgRNA targeting *rna* promoter	this study
pCRJ001-g028	pCRJ001 derivative expressing sgRNA targeting *rna* CDS	this study
pCRJ001-g030	pCRJ001 derivative expressing sgRNA targeting φ*T7 gene 3* CDS	this study
pCRJ001-g031	pCRJ001 derivative expressing sgRNA targeting *oxyS* promoter	this study
pCRJ001-g032	pCRJ001 derivative expressing sgRNA targeting *oxyS* CDS	this study
pCRJ001-g033	pCRJ001 derivative expressing sgRNA targeting *rne* promoter	this study
pCRJ001-g034	pCRJ001 derivative expressing sgRNA targeting *rne* CDS	this study
pCRJ001-g035	pCRJ001 derivative expressing sgRNA targeting *trxB* promoter	this study
pCRJ001-g036	pCRJ001 derivative expressing sgRNA targeting *trxB* CDS	this study
pCRJ001-g037	pCRJ001 derivative expressing nontargeting control sgRNA	this study
pBAD-trxA	pBAD derivative for *trxA* overexpression	this study
pBAD-trxB	pBAD derivative for *trxB* overexpression	this study
pBAD-mukB	pBAD derivative for *mukB* overexpression	this study
pBAD-infC	pBAD derivative for *infC* overexpression	this study
pBAD-rne	pBAD derivative for *rne* overexpression	this study
pBAD-oxyS	pBAD derivative for *oxyS* overexpression	this study
pBAD-cyaR	pBAD derivative for *cyaR* overexpression	this study
pBAD-dgt	pBAD derivative for *dgt* overexpression	this study

a sgRNA = single-guide RNA; CDS = coding sequence downstream of transcription start sites.

*E. coli* bacteriophage T7 (ATCC BAA-1025-B2) was propagated using *E. coli* BL21 as the host strain. BL21 was grown overnight (16 h) in 10 mL of φLB inoculated with a single colony. The overnight culture was transferred to 100 mL of prewarmed φLB in a 500 mL Erlenmeyer flask and shake-incubated for 1 h to bring the culture to the log phase. This culture was then inoculated with 100 μL of 10^8^ PFU/mL T7 lysate and allowed to incubate until complete bacterial lysis was visually observed. The lysate was allowed to shake and incubate a further 10 min after addition of 1 mL of chloroform to lyse remaining BL21 cells. The lysate was transferred to 50 mL conical tubes and centrifuged at 10,000*g* for 5 min to pellet cell debris and separate chloroform from the mixture. Supernatants were recovered, 0.22 μm sterile-filtered, and then placed on ice for 1 h. Phage were concentrated by PEG precipitation.^70^ Briefly, 0.22 μm sterile-filtered PEG-8000/NaCl solution was mixed with lysate supernatants to final concentrations of 4% (w/v) and 0.5 M, respectively, followed by overnight incubation at 4 °C to encourage precipitation. Phage were pelleted by centrifugation at 10,000*g* at 4 °C for 30 min. Supernatants were carefully decanted and discarded. Following a second centrifugation at 6000*g* at 4 °C for 1 min, residual PEG/NaCl solution was aspirated by pipet. Phage pellets were resuspended and consolidated in minimal SM buffer (50 mM Tris-Cl pH 7.5, 100 mM NaCl, 8 mM MgSO_4_). Phage stocks typically achieved titers of 10^12^–10^13^ PFU/mL and were stored at 4 °C.

### DNA Cloning and Preparation

Plasmids were purified using Qiagen kits, as described by the manufacturer. For cloning, plasmids were purified using Qiaprep Spin Miniprep Kits. Plasmids used in cell-free experiments were purified using Plasmid Plus Midi Kits. NEB DH5α was transformed with plasmids via heat-shock; BL21 was transformed via electroporation according to previously described protocols.^59^ CRISPRi crRNAs were designed to target experimentally confirmed promoter positions and transcription start sites using the more stringent TTTV PAM where possible and the less stringent TTV when not.^71^ CRISPRi constructs were all prepared using pCRJ001 as the vector backbone by replacing a LacZα with crRNA sequences via golden Gate cloning and X-gal screening as described.^31^ Overexpression vectors were prepared via NEB HiFI assembly per the manufacturer’s instructions. pBAD-sfGFP (Table 3) was used as the backbone for arabinose inducible gene expression. Plasmids were validated by Sanger sequencing by Azenta (South Plainfield, NJ). Transformants bearing sequence confirmed that plasmids were banked in glycerol stocks at −80 °C.

### Phage Genome Extraction

T7 genomes were extracted using Qiagen DNeasy Blood and Tissue Kits (Hilden, Germany) as previously described.^88^ Briefly, residual bacterial nucleic acids were removed from high titer lysates by 1 h incubation with nucleases at 37 °C: 450 μL of lysate, 50 μL of 10× nuclease buffer (100 mM Tris-Cl pH 7.5, 25 mM MgCl2, 1 mM CaCl2), 1 μL of 1 mg/mL DNase I (Sigma-Aldrich, St. Louis, MO), and 1 μL of 10 mg/mL RNase A (ThermoFisher Scientific, Roskilde, Denmark). Nucleases were inactivated by addition of 20 μL of 0.5 M ethylenediaminetetraacetic acid (EDTA) pH 8.0 and incubation at 70 °C for 10 min. Then, capsids were degraded by the addition of 1.25 μL of 20 mg/mL proteinase K and incubation at 56 °C for 1.5 h with gentle mixing by inversion every 30 min. Liberated T7 genomes were then purified following the Qiagen DNeasy Kit Protocols with one modification. Rather than eluting phage genomes from Qiagen affinity columns using the provided elution buffer, genomes were eluted using 55 °C nuclease-free water. DNA was quantified by using a DeNovix DS-11 microvolume spectrophotometer. Samples with 260/280 and 260/230 nm ratios from 1.8 to 2.0 were considered high-quality. Typical yields for a 10^12^ PFU/mL lysate were 100 μL of ∼500 to 2000 ng/μL genomic DNA (gDNA). DNA integrity was confirmed by gel electrophoresis. Purified T7 genomes were stored at −20 °C in 5 μL aliquots to minimize freeze/thaws. Aliquots were thawed on ice ∼30 min prior to use.

### Preparation of Gene Knockdown or Overexpressing Cultures for Phage Fitness Assays

*E. coli* BL21 carrying CRISPRi or overexpression plasmids was streaked from glycerol stocks onto φLB agar to generate isolated colonies. A single colony per construct was picked to inoculate overnight selective broth cultures which were then subcultured 1:10 into fresh broth and incubated 1 h to bring cells to the log phase. Gene repression or overexpression was induced by 1% inoculation of 1.2 mL of prewarmed φLB/cm + 2% rhamnose or φLB/amp + 0.2% arabinose with log-phase cultures, respectively. Induced cultures were incubated in 24-well plates (Corning Costar 3738 Not Treated) for at least 4 h until OD600 reached 0.4–0.6, then placed on ice for 15 min. Induced and noninduced controls were centrifuged at 6000*g* at 4 °C for 5 min, then resuspended in fresh media to OD = 0.3 and kept on ice until phage fitness assays (lysis timing and efficiency of plating). Nontargeting (NT)-controls were included in each CRISPRi experiment. Overexpression of sfGFP was included as a negative control in overexpression experiments. Biological replicates (*n* = 3) were performed on different days with cultures started from unique isolated colonies. Antibiotics and inducers were 0.22 μm filter-sterilized and added to broth cultures immediately before use.

### Phage Analytical Methods

T7 titers were determined using the standard double-layer agar (DLA) plaque assay [or DLA spot test] using BL21 as the host strain.^72^ Overnight cultures of BL21 in φLB were diluted 1:10 in prewarmed φLB and then incubated for 1 h to bring cultures to log-phase. Ten microliters [100 μL for spot test] of log-phase BL21 was mixed with 3 μL of appropriate dilutions of the T7 sample, incubated for 5 min, and then mixed with 1 mL [4 mL for the spot test] of 50 °C 0.7% SM agarose (SM buffer +0.7% agarose (w/v)) and poured over prewarmed φLB agar plates [for the spot test, samples were serially diluted in SM buffer and 3 μL was applied to the φLB agar plates]. The SM agarose was allowed to solidify at room temperature for 15 min, and then, plates were incubated at 37 °C for 1 h before overnight room temperature incubation. T7 titers were calculated from plaque counts [or triplicate spots]. Titers are given in plaque forming units/mL (PFU/mL).

Approximate phage titers were calculated using T7 promoter driven GFP as a proxy for phage concentration. Here, GFP production from *E. coli* BL21 carrying pJL1-sfGFP (T7 promoter-RBS-sfGFP-T7 terminator) was quantified using a microtiter plate reader (Spectramax iD5, Molecular Devices). Briefly, cells were prepared in the same manner as described above before being transferred to Corning black/clear-bottom 3631 nontreated microplates to 200 μL per well. The bacteria were then inoculated with 2 μL of phage samples, and infection kinetics tracked via OD600 and sfGFP fluorescence (485 nm excitation/515 emission) with “medium” orbital speed shaking between data collection. In vivo T7-mediated T7 RNA polymerase expression drives the sfGFP expression off the pJL1-sfGFP plasmid. Approximate T7 phage titers were calculated using a linear standard curve of known phage titers (1–10 log_10_ PFU/mL) versus time to *V*_max_ for relative fluorescence intensity with earlier *V*_max_ onset correlated with higher phage titers (Figure S5). Triplicate T7 standards, φLB, and bacteria without phage were included as controls in each assay plate. The limit of detection was 10 PFU/mL detectible within 2 h of inoculation.

Efficiency of plating (EOP) assay was performed to determine the ratio of plaque counts between samples. Briefly, 50 μL of knockdown and overexpressing BL21 strains at OD600 = 0.3 were infected with T7 at an MOI = 0.0001 and incubated while shaking for 30 min. After incubation, 100 μL of ice-cold chloroform was added to each well to halt phage replication and lyse the remaining intact cells. Samples were transferred to microcentrifuge tubes containing 500 μL of ice-cold SM buffer and centrifuged at 17,000*g* for 1 min for phase separation. The supernatants were further diluted in SM buffer, as necessary. Phage titers were counted by spot tests on wild-type BL21 overlay plates from the average of three 3 μL technical replicate spots. Noninduced knockdown and overexpressing strains were included as controls.

### Gene Transcription Analysis

RNA extractions were performed using Qiagen RNeasy Kit with enzymatic lysis and an additional DNase treatment as previously described.^73^ Log-phase cells (0.5 mL, OD600 = 0.4–0.6) were pelleted and resuspended in 200 μL of lysis buffer pH 8.5 (15 mg/mL lysozyme, 1 mg/mL proteinase K, 30 mM Tris-Cl, 1 mM EDTA) and then incubated at room temperature for 10 min with gentle vortexing every 2 min. The lysate was processed using the standard Qiagen RNeasy Kit protocol including on-column DNase I treatment and eluted using 30 μL of nuclease-free water. A second DNase treatment was performed using a 30 μL reaction volume containing 26 μL of RNA extract, 3 μL 10× TURBO DNase Buffer, and 1 μL of TURBO DNase incubated at 37 °C for 30 min. DNase activity was stopped using 3 μL of DNase Inactivation reagent. RNA was quantified by using a DeNovix DS-11 microvolume spectrophotometer. Samples with 260/280 and 260/230 nm ratios from 2 to 2.2 were considered high-quality RNA. cDNA was generated using SuperScript II Reverse Transcriptase with RNA normalized to 50 ng per reaction. RT-qPCR was performed in 20 μL reaction containing 10 μL iTaq Universal SYBR Green Supermix (2×) (Bio-Rad, Hercules, CA), 500 nM forward and reverse primers (Table S1), and 1 ng cDNA. Relative mRNA expression was calculated using the 2^–ΔΔCt^ method.^74^ Expression of *cysG/hcaT/idnT* was averaged to be used as references genes due to their stable expression in the context of induced protein expression in *E. coli* BL21.^75^

### Preparation of Cell Extracts for CFES and CFBS

Cell extracts were prepared based on Kwon and co-workers with modification.^76^ Each knockdown and overexpression strain was grown overnight in LB with the appropriate antibiotic from a single colony. Overnight cultures were diluted 1:10 in prewarmed selective LB and incubated for 1 h to bring the cultures to the log-phase, which were then diluted 1:100 in selective prewarmed 2 × YTP media (16 g/L of tryptone, 10 g/L of yeast extract, 5 g/L of sodium chloride, 7 g/L of potassium phosphate dibasic, 3 g/L of potassium phosphate monobasic, pH 7.2) and incubated until early exponential phage (OD600 = 0.2) where inducers L-rhamnose or l-arabinose were added to 2 and 0.2% as appropriate. When the OD600 reached 2.0–3.0, flasks were rapidly chilled on ice for 15 min and remained cold for the rest of the extraction. Cultures were then centrifuged at 10,000 *g* for 5 min at 4 °C. The supernatants were removed, and pellets were washed three times with ice-cold S30B buffer (10 mM Tris-Cl, pH 8.2, 14 mM magnesium glutamate, 60 mM potassium glutamate, 2 mM DTT). The pellet wet-mass (g) was recorded followed by flash-freezing in liquid nitrogen and storage at −80 °C overnight. Frozen pellets were thawed slowly in ice water and then resuspended in S30B (0.8 mL per g of wet-mass) and transferred to 1.5 mL microcentrifuge tubes with final volumes 500–1000 μL. Cell slurries were lysed in an ice–water bath using the Qsonica Q125 Sonicator with 1/8 in. probe set to 50% amplitude with 10s on/off pulses to avoid overheating samples. Target total energy input was calculated using eq 1:^66^

DTT was added to each lysate for a final concentration of 3 mM concentration. Cell-lysates were centrifuged at 12,000*g* for 10 min at 4 °C. Supernatants from each strain were consolidated, then incubated at 37 °C shaking for 80 min for a runoff reaction followed by another centrifugation at 10,000*g* for 10 min at 4 °C. Finally, these clarified supernatants were aliquoted and flash-frozen with liquid nitrogen then stored at −80 °C. Total protein was measured using the Pierce BCA Protein Assay Kit (ThermoFisher) using bovine serum albumin standards. The presence of residual bacteria was checked by spotting clarified lysates on LB agar and incubating overnight. Inducers were dissolved in 2xYTP and 0.22 μm filter-sterilized. Biological duplicates were prepared for each selected knockdown and overexpression strain. Extracts based on wild-type BL21 absent plasmids were prepared as baseline references for protein and phage synthesis.

### CFES TXTL Protein Synthesis Reporter Assay (T7 gDNA + pJL1-sfGFP Cascade)

Activity of cell extracts for T7 gDNA-dependent transcription and protein synthesis was evaluated by using sfGFP synthesis as a reporter. Protein synthesis was performed in 15 μL reactions incubated at 30 °C in 1.5 mL tubes with cell extracts occupying one-third of the volume and reaction buffer and DNA templates the remaining two-thirds. Reactions contained 57 mM HEPES, pH 8, 130 mM K(glu), 12 mM Mg(glu)_2_, 0.4 mM NAD, 0.27 mM CoA, 0.75 mM cAMP, 2 mM spermidine, 1 mM DTT, 1.5 mM ATP and GTP, 0.9 mM CTP and UTP, 0.2 mg/mL *E. coli* tRNA, 0.068 mM folinic acid, 2 mM of each canonical amino acid except glutamate, 33 mM PEP, 12.66 mg/mL maltodextrin, 0.5 nM T7 gDNA, and 5 nM pJl1-sfGFP. Reaction components were mixed and then DNA added last before being placed on ice for 5 min prior to incubation at 30 °C. Five microliter samples were taken at 4 and 20 h, diluted in 20 μL of 50 mM HEPES, pH 8, and sfGFP fluorescence measured as described above in Greiner Bio-One #781209 black flat-bottom 384-well plates. All reactions were run in triplicate.

### CFBS for Bacteriophage Synthesis

Bacteriophage synthesis was performed using the reaction setup described above with modifications: pJL1-sfGFP was omitted, and reaction buffer was supplemented with 3.5% (w/v) PEG-8000 and 0.5 mM dNTPs. Purified T7 genomic DNA was used as a template at 0.5 nM. At 4 and 20 h, 3 μL samples were taken and diluted in 30 μL of SM buffer. Approximate T7 titers were calculated by the rapid titer estimation assay described above. More precise titers were calculated from plaque counts by using the DLA method.

## Supporting Information Available

DNA sequence information; concept art; plasmid design; and experimental workflow (PDF)

## Abbreviations

CFES: Cell-free expression system
CFBS: Cell-free bacteriophage synthesis
TXTL: transcription/translation
CRISPRi: CRISPR interference
KD: knockdown
OX: overexpression
PFU: plaque forming units
EOP: efficiency of plating
MOI: multiplicity of infection
crRNA: single-guide RNA
TSS: transcription start site
T7gp3: T7 exonuclease gene 3
OD: optical density.
