# Supplementary material for: Cell Free Bacteriophage Synthesis from Engineered Strains Improves Yield
Source: ACS Synth Biol. 2023 Aug 7;12(8):2418–31. doi: 10.1021/acssynbio.3c00239 (PMC10443043; doi:10.1021/acssynbio.3c00239)
Supplement: Supplementary file 1 — sb3c00239_si_001.pdf [file sb3c00239_si_001.pdf]

Supporting Information

Cell Free Bacteriophage Synthesis from Engineered Strains Improves Yield

Rani T Brooks[1], Lisa Morici[2], and Nicholas R Sandoval[3†]

[1] Interdisciplinary Bioinnovation PhD Program, Tulane University, New Orleans, LA, United States

[2] Department of Microbiology and Immunology, Tulane University School of Medicine, 70112, New Orleans, LA, United States

[3] Department of Chemical & Biomolecular Engineering, Tulane University, New Orleans, LA, United States

[†]Corresponding author

Table S1. Primers and DNA oligos used in this study.

Name	Sequence	Description
g001 top	agatcgaaagcgtatccggtgaaa	guide RNA Goldengate insert top strand targeting trxA promoter
g001 bottom	atactttcaccggatacgctttcg	guide RNA Goldengate insert bottom strand targeting trxA promoter
g002 top	agattatatgagcgataaaattat	guide RNA Goldengate insert top strand targeting trxA CDS
g002 bottom	atacataattttatcgctcatata	guide RNA Goldengate insert bottom strand targeting trxA CDS
g003 top	agattggttcgcctggtatccttt	guide RNA Goldengate insert top strand targeting nusG promoter
g003 bottom	atacaaaggataccaggcgaacca	guide RNA Goldengate insert bottom strand targeting nusG promoter
g004 top	agataaggccgcgtagcaacgtcg	guide RNA Goldengate insert top strand targeting nusG CDS
g004 bottom	ataccgacgttgctacgcggcctt	guide RNA Goldengate insert bottom strand targeting nusG CDS
g005 top	agatctgcaaaaagcagcacaagg	guide RNA Goldengate insert top strand targeting pgk promoter
g005 bottom	atacccttgtgctgctttttgcag	guide RNA Goldengate insert bottom strand targeting pgk promoter
g006 top	agatagatgaccgatctggatctt	guide RNA Goldengate insert top strand targeting pgk CDS
g006 bottom	atacaagatccagatcggtcatct	guide RNA Goldengate insert bottom strand targeting pgk CDS
g007 top	agattctttcttcgtctaaagatt	guide RNA Goldengate insert top strand targeting subH(ssyA) promoter
g007 bottom	atacaatctttagacgaagaaaga	guide RNA Goldengate insert bottom strand targeting subH(ssyA) promoter
g008 top	agatgtgaccaacgtagataaagc	guide RNA Goldengate insert top strand targeting subH(ssyA) CDS
g008 bottom	atacgctttatctacgttggtcac	guide RNA Goldengate insert bottom strand targeting subH(ssyA) CDS
g009 top	agattgccagactgtaagtacccg	guide RNA Goldengate insert top strand targeting eno promoter
g009 bottom	ataccgggtacttacagtctggca	guide RNA Goldengate insert bottom strand targeting eno promoter
g010 top	agataagccgaagtacatctggag	guide RNA Goldengate insert top strand targeting eno CDS
g010 bottom	atacctccagatgtacttcggctt	guide RNA Goldengate insert bottom strand targeting eno CDS

g011 top	agattcggaactggatatgatggt	guide RNA Goldengate insert top strand targeting mukB promoter
g011 bottom	atacaccatcatatccagttccga	guide RNA Goldengate insert bottom strand targeting mukB promoter
g012 top	agatgctcactgacgctgattaac	guide RNA Goldengate insert top strand targeting mukB CDS
g012 bottom	atacgttaatcagcgtcagtgagc	guide RNA Goldengate insert bottom strand targeting mukB CDS
g013 top	agataaatgcacaaaacttttata	guide RNA Goldengate insert top strand targeting hemL promoter
g013 bottom	atactataaaagttttgtgcattt	guide RNA Goldengate insert bottom strand targeting hemL promoter
g014 top	agatcagcgcagcgcgcgagctga	guide RNA Goldengate insert top strand targeting hemL CDS
g014 bottom	atactcagctcgcgcgctgcgctg	guide RNA Goldengate insert bottom strand targeting hemL CDS
g015 top	agatttaaggttatcatccgtttc	guide RNA Goldengate insert top strand targeting cyaR (ryeE) promoter
g015 bottom	atacgaaacggatgataaccttaa	guide RNA Goldengate insert bottom strand targeting cyaR (ryeE) promoter
g016 top	agatgcctgtgtaatctcccttac	guide RNA Goldengate insert top strand targeting cyaR (ryeE) CDS
g016 bottom	atacgtaagggagattacacaggc	guide RNA Goldengate insert bottom strand targeting cyaR (ryeE) CDS
g017 top	agatctgtatatactcacagcata	guide RNA Goldengate insert top strand targeting lexA promoter
g017 bottom	atactatgctgtgagtatatacag	guide RNA Goldengate insert bottom strand targeting lexA promoter
g018 top	agatatctcatccgtgatcacatc	guide RNA Goldengate insert top strand targeting lexA CDS
g018 bottom	atacgatgtgatcacggatgagat	guide RNA Goldengate insert bottom strand targeting lexA CDS
g019 top	agatgctttaaaatccgcgagcac	guide RNA Goldengate insert top strand targeting infC promoter
g019 bottom	atacgtgctcgcggattttaaagc	guide RNA Goldengate insert bottom strand targeting infC promoter
g020 top	agattcgtataatggattacggca	guide RNA Goldengate insert top strand targeting infC CDS
g020 bottom	atactgccgtaatccattatacga	guide RNA Goldengate insert bottom strand targeting infC CDS
g021 top	agatcgctagtttcacccgggggc	guide RNA Goldengate insert top strand targeting recC promoter

g021 bottom	atacgcccccgggtgaaactagcg	guide RNA Goldengate insert bottom strand targeting recC promoter
g022 top	agatttgtcgaacgcgaacggctg	guide RNA Goldengate insert top strand targeting recC CDS
g022 bottom	ataccagccgttcgcgttcgacaa	guide RNA Goldengate insert bottom strand targeting recC CDS
g023 top	agataaggcatagtttaccatgcg	guide RNA Goldengate insert top strand targeting dgt promoter
g023 bottom	ataccgcatggtaaactatgcctt	guide RNA Goldengate insert bottom strand targeting dgt promoter
g024 top	agatcactggagcgcaatgccgcc	guide RNA Goldengate insert top strand targeting dgt CDS
g024 bottom	atacggcggcattgcgctccagtg	guide RNA Goldengate insert bottom strand targeting dgt CDS
g025 top	agatgactggtatcaggcggatga	guide RNA Goldengate insert top strand targeting udk promoter
g025 bottom	atactcatccgcctgataccagtc	guide RNA Goldengate insert bottom strand targeting udk promoter
g026 top	agattcgtgaattacgtgagcaag	guide RNA Goldengate insert top strand targeting udk CDS
g026 bottom	ataccttgctcacgtaattcacga	guide RNA Goldengate insert bottom strand targeting udk CDS
g027 top	agattgatttaatagatagtattc	guide RNA Goldengate insert top strand targeting rna promoter
g027 bottom	atacgaatactatctattaaatca	guide RNA Goldengate insert bottom strand targeting rna promoter
g028 top	agatatcgctatgtcctggccctc	guide RNA Goldengate insert top strand targeting rna CDS
g028 bottom	atacgagggccaggacatagcgat	guide RNA Goldengate insert bottom strand targeting rna CDS
g029 top	agatggctaccgtagagtcctcgg	guide RNA Goldengate insert top strand targeting gp2 CDS
g029 bottom	atacttgggctaccgtagagtcctcgg	guide RNA Goldengate insert bottom strand targeting gp2 CDS
g030 top	agatgctctggcctagaggacaag	guide RNA Goldengate insert top strand targeting gp2 CDS
g030 bottom	ataccttgtcctctaggccagagc	guide RNA Goldengate insert bottom strand targeting gp2 CDS
g031 top	agattccctatcaagcattctgac	guide RNA Goldengate insert top strand targeting oxys
g031 bottom	atacGTCAGAATGCTTGATAGGGA	guide RNA Goldengate insert bottom strand targeting oxys

g032 top	agatacccttgaagtcactgcccg	guide RNA Goldengate insert top strand targeting oxys
g032 bottom	ataccgggcagtgacttcaagggt	guide RNA Goldengate insert bottom strand targeting oxys
g033 top	agatcagtggaataatgaggccgt	guide RNA Goldengate insert top strand targeting rne promoter
g033 bottom	atacacggcctcattattccactg	guide RNA Goldengate insert bottom strand targeting rne promoter
g034 top	agatttgattacggcgctgaacgt	guide RNA Goldengate insert top strand targeting rne CDS
g034 bottom	atacacgttcagcgccgtaatcaa	guide RNA Goldengate insert bottom strand targeting rne CDS
g035 top	agatcgtctgtaaattccctacaa	guide RNA Goldengate insert top strand targeting trxB promoter
g035 bottom	atacttgtagggaatttacagacg	guide RNA Goldengate insert bottom strand targeting trxB promoter
g036 top	agataaactgagatcatttttgat	guide RNA Goldengate insert top strand targeting trxB CDS
g036 bottom	atacatcaaaaatgatctcagttt	guide RNA Goldengate insert bottom strand targeting trxB CDS
g037 top	agatGCACACTGGGCAATACGAGG	guide RNA Goldengate insert top strand targeting NT-control
g037 bottom	atacCCTCGTATTGCCCAGTGTGC	guide RNA Goldengate insert bottom strand targeting NT-control
hcat FWD	CGCTCGGCTATTTCACATACT	RT-qPCR reference gene primer
hcat REV	GGTTTCTGGCGTTAAACCAATC	RT-qPCR reference gene primer
cysG FWD	CCGTCTGGTTTCTGACGATATT	RT-qPCR reference gene primer
cysG REV	CGCAGCAGGATCTGGTTAAT	RT-qPCR reference gene primer
idnT FWD	GTGCGCCTCTTCTTTGAATTT	RT-qPCR reference gene primer
idnT REV	TCGATGGTGCGTCCATTAC	RT-qPCR reference gene primer
trxA FWD	AATATGGCATCCGTGGTATCC	RT-qPCR primer
trxA REV	GTTAGCGTCGAGGAACTCTTT	RT-qPCR primer
FndCas12a FWD	CCTGTTCGATGAGAGGAATCTG	RT-qPCR primer
FndCas12a REV	CCTTAGCTGGGTGAGTGATTT	RT-qPCR primer

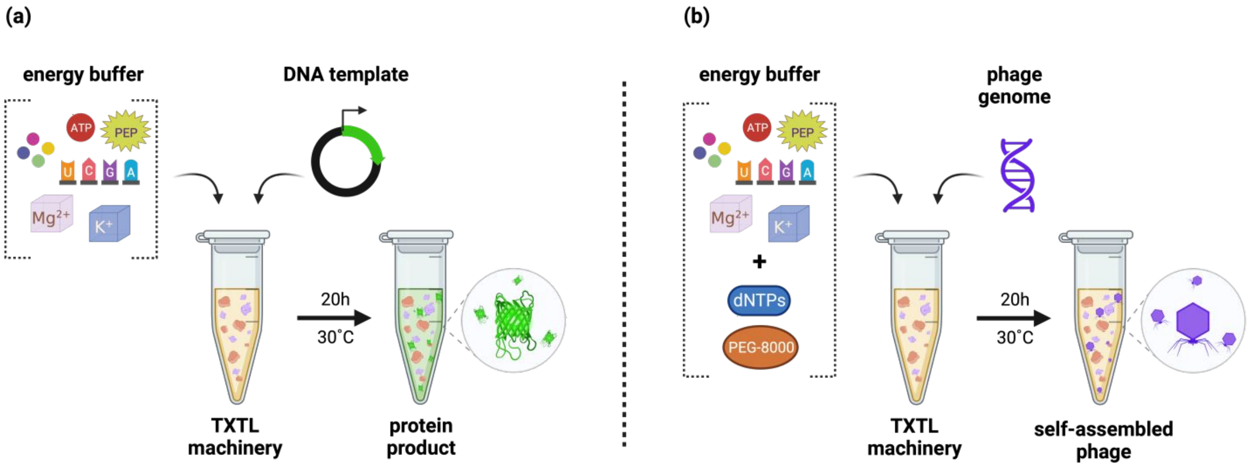

Figure S1. Comparison of cell-free systems for protein or phage synthesis. In vitro synthesis of (a) fluorescent reporter protein sfGFP and cell-free bacteriophage synthesis (b) of phage T7. Each system contains transcription/translation (TXTL) machinery derived from *E. coli* BL21 lysates, a DNA template, and energy buffer (20 canonical amino acids, PEP, NTPs, cofactors, and coenzymes). sfGFP in encoded on a circular plasmid (a) and phage T7 expression encoded on purified T7 gDNA. CFBS reactions are supplemented with molecular crowder PEG-8000 and dNTPs to support genome replication.

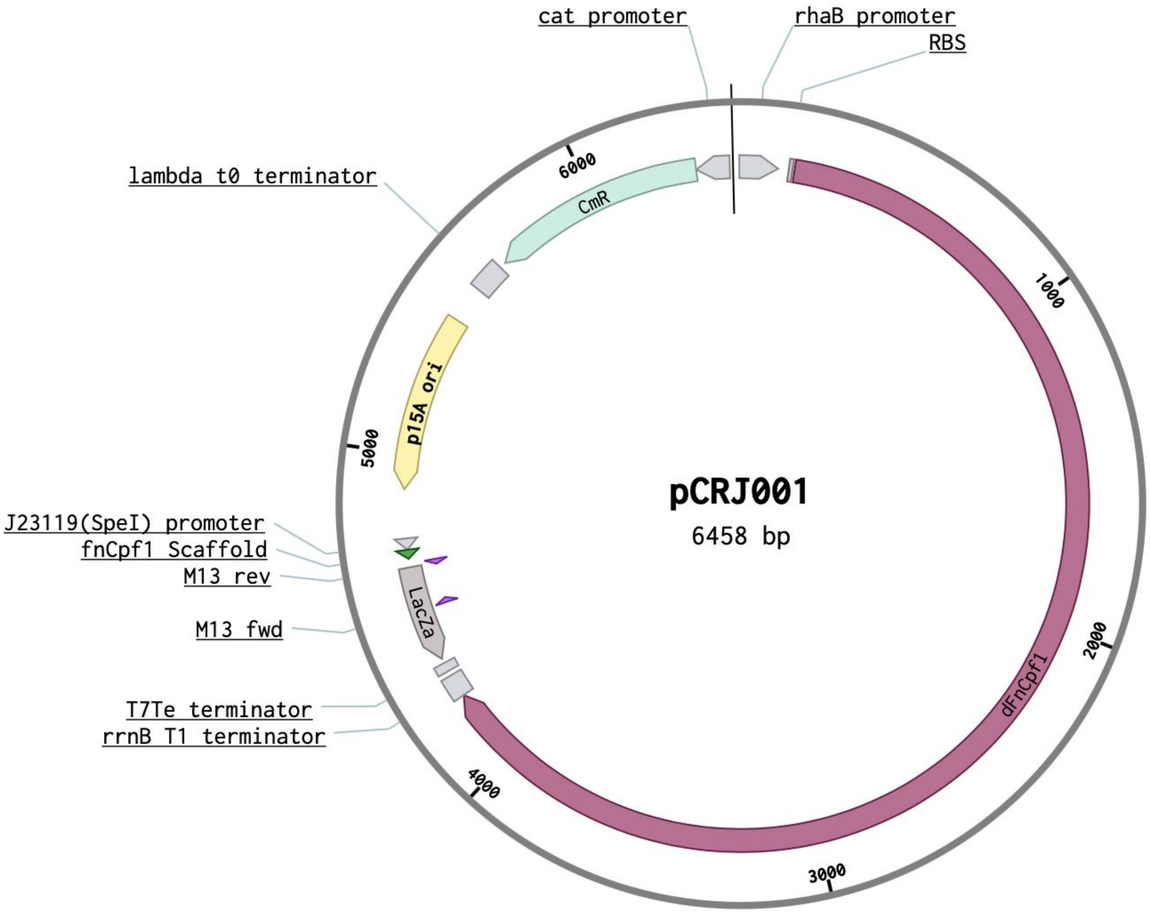

Figure S2. Single-plasmid CRISPR interference (CRISPRi) vector pCRJ001.

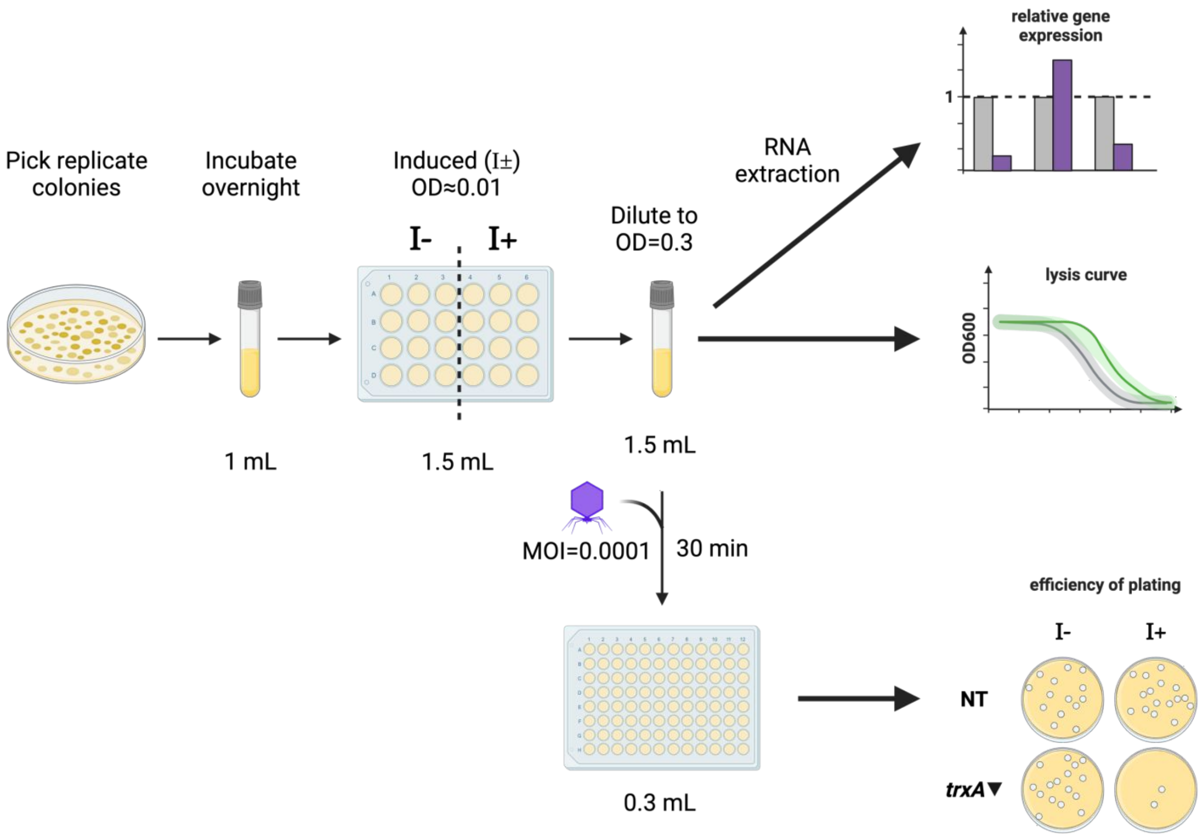

Figure S3. CRISPRi/overexpression induction experimental workflow. Phenotypic readouts for gene repression and overexpression on T7 fitness included impacts on lysis curve profile (e.g. lysis timing, mean lysis timing) and efficiency of plating. Gene repression was validated by RT-qPCR.

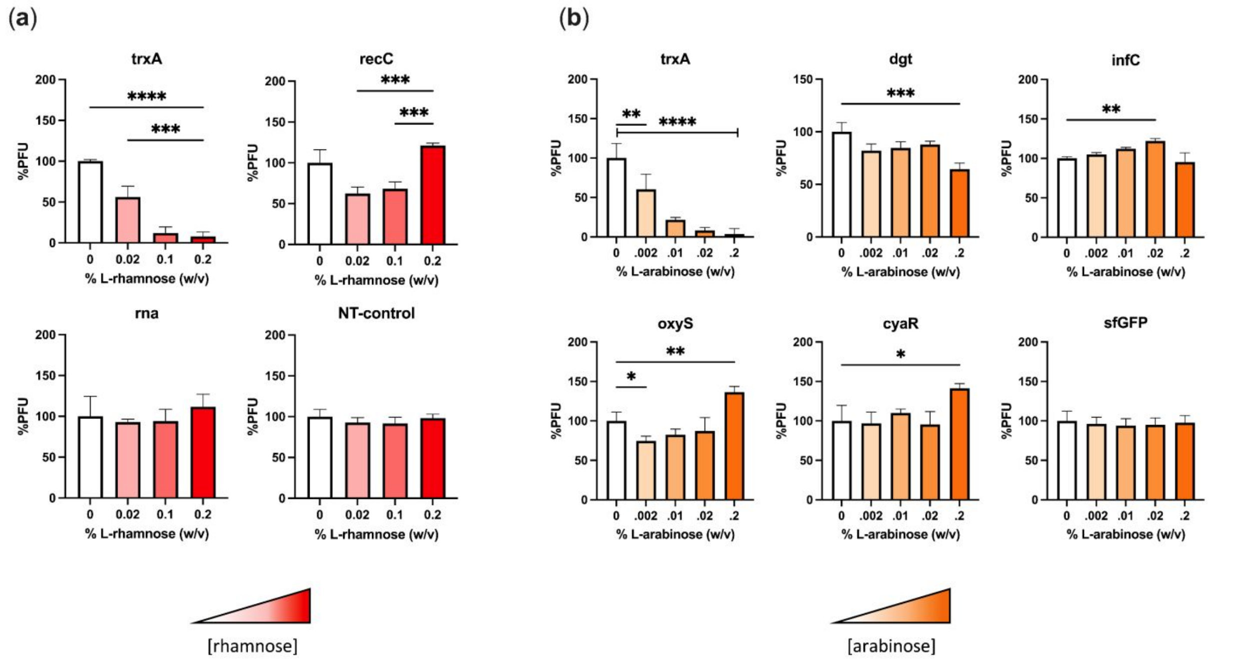

Figure S4. CRISPRi (KD) and overexpression (OX) inducer titration. CRISPRi knockdowns (KD) were induced in strains carrying pCRJ001 vectors using 0.02, 0.1, and 0.2% (w/v) L-rhamnose (**a**) and overexpression from pBAD vectors induced using 0.002, 0.01, 0.02, and 0.2% (w/v) L-arabinose (**b**). Bars reflects efficiency of plating (EOP) from 30 min T7 infections (MOI = 0.0001). Data represented as mean ± SD (n=3). Ordinary One-way ANOVAs were performed indicating significant differences in EOP ($p<0.05$). A non-targeting control (NT-control) was included for KD strains and pBAD-sfGFP for OX strains.

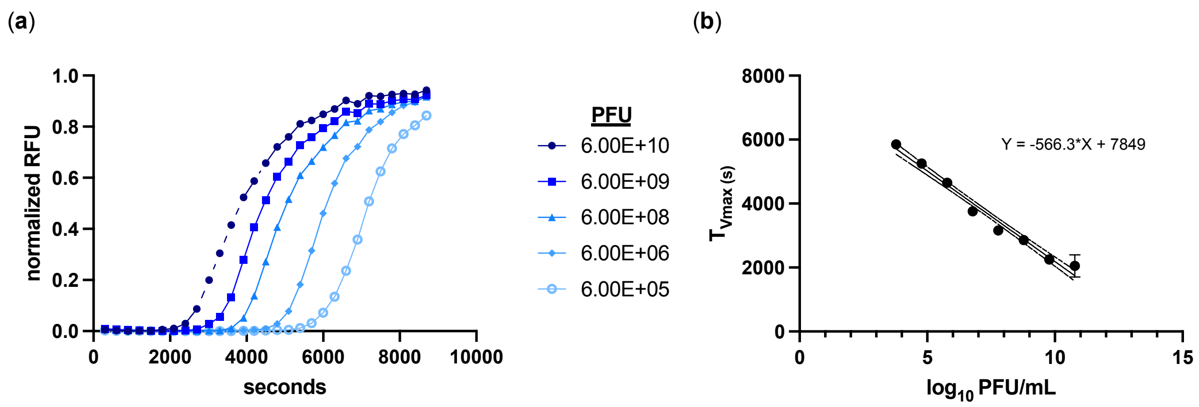

Figure S5. Rapid T7 enumeration assay. T7 infected log-phase BL21 carrying pJl1-T7-Pr-sfGFP. T7 RNA polymerase expressed during T7 infection drives sfGFP expression from the plasmid. Timing of sfGFP expression was dependent on T7 MOI (**a**) measured by normalized relative fluorescence units (RFU). A standard curve of log phage titer vs time to maximum sfGFP expression rate (T_{Vmax}) (**b**) was generated and used to rapidly estimate T7 titers in cell-free bacteriophage synthesis experiments to inform dilutions for plaque assays. Dotted lines represent 95% CI for simple linear regression.
